# *catena*-Poly[[diaqua­strontium]-bis­(μ-quinoline-3-carboxyl­ato)]

**DOI:** 10.1107/S1600536811036610

**Published:** 2011-09-14

**Authors:** Dong-Liang Miao, Shi-Jie Li, Wen-Dong Song, Xiao-Tian Ma, Xiao-Fei Li

**Affiliations:** a College of Food Science and Technology, Guangdong Ocean University, Zhanjiang 524088, People’s Republic of China; b School of Environmental Science and Engineering, Donghua University, Shanghai 200051, People’s Republic of China; c College of Science, Guangdong Ocean University, Zhanjiang 524088, People’s Republic of China; d College of Agronomy, Guangdong Ocean University, Zhanjiang 524088, People’s Republic of China

## Abstract

The title compound, [Sr(C_10_H_6_NO_2_)_2_(H_2_O)_2_]_*n*_, contains an eight-coordinate Sr^II^ ion displaying a distorted square-anti­prismatic geometry, two quinoline-3-carboxyl­ate ligands and two terminal water mol­ecules. The Sr^II^ atom is surrounded by six carboxyl­ate O atoms from four separate quinoline-3-carboxyl­ate ligands and two O atoms from two coordinated water mol­ecules. The bridging carboxyl­ate O atoms [Sr—O = 2.498 (3) and 2.495 (3) Å] link Sr^II^ atoms, forming a chain substructure extending along the *c* axis. The chains are linked by O—H⋯N and O—H⋯O hydrogen bonds, giving a three-dimensional framework structure

## Related literature

For a similar structure, see: Miao *et al.* (2010 ▶). For structures with quinoline-3-carboxyl­ate ligands, see: Okabe & Muranishi (2003*a* ▶,*b* ▶); Zevaco *et al.* (1998 ▶). For quinoline-3-carboxyl­ate ligands in a range of metal complexes, see: Haendler (1986 ▶, 1996 ▶); Hu *et al.* (2007 ▶); Martell & Smith (1974 ▶); Odoko *et al.* (2001 ▶); Okabe & Koizumi (1997 ▶); Okabe & Makino (1998 ▶, 1999 ▶); Okabe & Muranishi (2002 ▶).
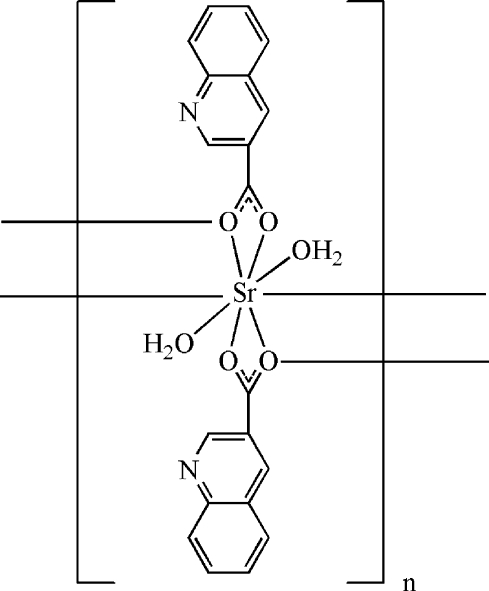


## Experimental

### 

#### Crystal data

[Sr(C_10_H_6_NO_2_)_2_(H_2_O)_2_]
*M* *_r_* = 467.97
Monoclinic, 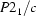

*a* = 16.121 (3) Å
*b* = 15.568 (3) Å
*c* = 7.9607 (16) Å
β = 97.42 (3)°
*V* = 1981.2 (7) Å^3^
*Z* = 4
Mo *K*α radiation
μ = 2.76 mm^−1^
*T* = 293 K
0.30 × 0.28 × 0.22 mm

#### Data collection

Bruker APEXII area-detector diffractometer
Absorption correction: multi-scan (*SADABS*; Bruker, 2001 ▶) *T*_min_ = 0.491, *T*_max_ = 0.582
15104 measured reflections
3551 independent reflections
2571 reflections with *I* > 2σ(*I*)
*R*_int_ = 0.047

#### Refinement

*R*[*F* ^2^ > 2σ(*F* ^2^)] = 0.037
*wR*(*F* ^2^) = 0.112
*S* = 1.19
3551 reflections
274 parameters
6 restraints
H atoms treated by a mixture of independent and constrained refinement
Δρ_max_ = 0.79 e Å^−3^
Δρ_min_ = −1.19 e Å^−3^

### 

Data collection: *APEX2* (Bruker, 2004 ▶); cell refinement: *SAINT* (Bruker, 2002 ▶); data reduction: *SAINT*; program(s) used to solve structure: *SHELXS97* (Sheldrick, 2008 ▶); program(s) used to refine structure: *SHELXL97* (Sheldrick, 2008 ▶); molecular graphics: *SHELXTL* (Sheldrick, 2008 ▶); software used to prepare material for publication: *SHELXL97*.

## Supplementary Material

Crystal structure: contains datablock(s) I, global. DOI: 10.1107/S1600536811036610/jh2325sup1.cif

Structure factors: contains datablock(s) I. DOI: 10.1107/S1600536811036610/jh2325Isup2.hkl

Additional supplementary materials:  crystallographic information; 3D view; checkCIF report

## Figures and Tables

**Table 1:** Hydrogen-bond geometry (Å, °)

*D*—H⋯*A*	*D*—H	H⋯*A*	*D*⋯*A*	*D*—H⋯*A*
O1*W*—H2*W*⋯O2^i^	0.84 (1)	1.97 (2)	2.798 (5)	168 (6)
O1*W*—H1*W*⋯N1^ii^	0.84 (1)	2.01 (1)	2.846 (6)	175 (6)
O2*W*—H3*W*⋯O3^iii^	0.84 (1)	1.99 (2)	2.810 (5)	166 (5)
O2*W*—H4*W*⋯N2^iv^	0.84 (1)	2.01 (1)	2.846 (6)	176 (5)

Symmetry codes: (i) 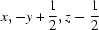; (ii) 
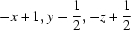
; (iii) 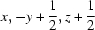; (iv) 
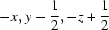
.

**Fig. 1.**
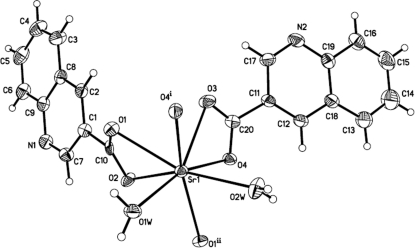
The structure of the title compound, showing the atomic numbering scheme. Non-H atoms are shown with 30% probability displacement ellipsoids.

**Fig. 2.**
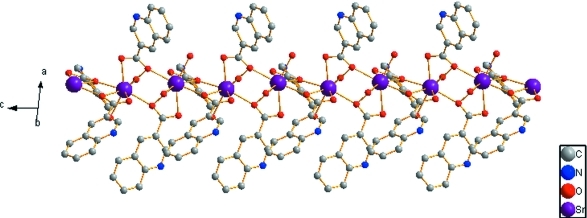
The one-dimensional chain substructure of (I) extending along the c axis.

**Fig. 3.**
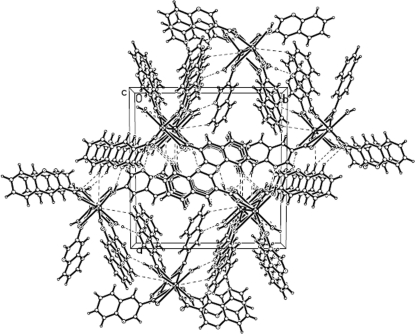
The three-dimensional hydrogen-bonded structure of (I).

## supplementary crystallographic information

### Comment

Crystal engineering of mental-organic complexes is a very active research field. It is well known that organic ligands play a crucial role in the design and construction of desirable frameworks. Quinoline-2-carboxylic acid is a tryptophan metabolite and it is known to be a chelator of transition metal ions (Martell & Smith, 1974). The crystal structures of its metal complexes have been determined for several metal ions, including Fe^II^ (Okabe & Makino, 1998; Okabe & Muranishi (2003a), Zn^II^ (Zevaco *et al.*, 1998; Okabe & Muranishi (2003b), Ni^II^ (Odoko *et al.*, 2001), V^IV^ (Okabe & Muranishi, 2002), Cu^II^ (Haendler, 1986), Mn^II^ (Haendler, 1986; Okabe & Koizumi, 1997) and Co^II^ (Okabe & Makino, 1999). However, to the best of our knowledge, the complexes based on the quinoline-3-carboxylate ligand are still largely unexplored(Hu *et al.*, 2007), In our previous study, we obtained a new Ca^II^ complex with quinoline-3-carboxylate ligand (Miao *et al.*, 2010). In this paper, we will present the synthesis and crystal structure of a new Sr(II) complex assembled from SrCl_2_ and quinoline-3-carboxylate ligand.

As illustrated in Fig. 1, the title complex [Sr(C_10_H_6_NO_2_)_2_ (H_2_O)_2_]_n_, contains a eight-coordinate Sr^II^ ion, two quinoline-3-carboxylate ligands and two terminal water molecules. Each Sr^II^ displays a distorted square-antiprismatic geametry defined by six carboxylate O atoms, from four separate quinoline-3-carboxylate ligands and two oxygen atoms from two aqual ligands. It is noted that the quinoline-3-carboxylate only one coordination mode in the title complex: each adopts bidentate chelating and bridging coordination fashion to connect two adjacent Sr^II^ ions. The bridging carboxylate O atoms (O1 and O4) [Sr—O, 2.498 (3), 2.495 (3) Å] link separate Sr^II^ centres, forming a one-dimensional chain substructure extended along *c* (Fig.2). The chains are linked together by O—H···N and O—H···O hydrogen bonds (Table 1) giving a three-dimensional framework structure (Fig.3).

### Experimental

A mixture of SrCl_2_ (0.05 g, 0.2 mmol) and quinoline-3-carboxylic acid (0.04 g, 0.2 mmol) in 12 ml of distilled water was sealed in an autoclave equipped with a Teflon liner (20 ml) and then heated at 394 K for 2 days. Crystals of the title compound were obtained by slow evaporation of the solvent at room temperature.

### Refinement

Water H atoms were located in a difference Fourier map and were allowed to ride on the parent atom, with *U*_iso_(H) = 1.5*U*_eq_(O). Carboxyl H atoms were located in a difference map and refined with distance restraints, *U*_iso_(H) = 1.5*U*_eq_(O). Other H atoms were placed at calculated positions and were treated as riding on parent atoms with C—H = 0.96 (methyl), 0.97 (methylene) and N—H = 0.86 Å, *U*_iso_(H) = 1.2 or 1.5*U*_eq_(C,*N*). The propyl groups of H_3_pimda are disordered over two sites with refined occupancies of 0.768 (6):0.232 (6) and 0.642 (8):0.358 (8). C—C distance restraints of disordered components were applied. The O3W water molecule is located close to an inversion center, its occupancy factor was refined to 0.49 (1) and was fixed as 0.5 at the final refinements.

### Crystal data

**Table:**

[Sr(C_10_H_6_NO_2_)_2_(H_2_O)_2_]	*Z* = 4
*M**_r_* = 467.97	*F*(000) = 944
Monoclinic, *P*2_1_/*c*	*D*_x_ = 1.569 Mg m^−^^3^
Hall symbol: -P 2ybc	Mo *K*α radiation, λ = 0.71073 Å
*a* = 16.121 (3) Å	µ = 2.76 mm^−^^1^
*b* = 15.568 (3) Å	*T* = 293 K
*c* = 7.9607 (16) Å	Block, colorless
β = 97.42 (3)°	0.30 × 0.28 × 0.22 mm
*V* = 1981.2 (7) Å^3^	

### Data collection

**Table:**

Bruker APEXII area-detector diffractometer	3551 independent reflections
Radiation source: fine-focus sealed tube	2571 reflections with *I* > 2σ(*I*)
graphite	*R*_int_ = 0.047
φ and ω scan	θ_max_ = 25.2°, θ_min_ = 3.0°
Absorption correction: multi-scan (*SADABS*; Bruker, 2001)	*h* = −19→19
*T*_min_ = 0.491, *T*_max_ = 0.582	*k* = −18→18
15104 measured reflections	*l* = −9→9

### Refinement

**Table:**

Refinement on *F*^2^	Primary atom site location: structure-invariant direct methods
Least-squares matrix: full	Secondary atom site location: difference Fourier map
*R*[*F*^2^ > 2σ(*F*^2^)] = 0.037	Hydrogen site location: inferred from neighbouring sites
*wR*(*F*^2^) = 0.112	H atoms treated by a mixture of independent and constrained refinement
*S* = 1.19	*w* = 1/[σ^2^(*F*_o_^2^) + (0.0192*P*)^2^ + 6.2168*P*] where *P* = (*F*_o_^2^ + 2*F*_c_^2^)/3
3551 reflections	(Δ/σ)_max_ < 0.001
274 parameters	Δρ_max_ = 0.79 e Å^−^^3^
6 restraints	Δρ_min_ = −1.19 e Å^−^^3^

### Special details

**Table:**

Geometry. All e.s.d.'s (except the e.s.d. in the dihedral angle between two l.s. planes) are estimated using the full covariance matrix. The cell e.s.d.'s are taken into account individually in the estimation of e.s.d.'s in distances, angles and torsion angles; correlations between e.s.d.'s in cell parameters are only used when they are defined by crystal symmetry. An approximate (isotropic) treatment of cell e.s.d.'s is used for estimating e.s.d.'s involving l.s. planes.
Refinement. Refinement of *F*^2^ against ALL reflections. The weighted *R*-factor *wR* and goodness of fit *S* are based on *F*^2^, conventional *R*-factors *R* are based on *F*, with *F* set to zero for negative *F*^2^. The threshold expression of *F*^2^ > σ(*F*^2^) is used only for calculating *R*-factors(gt) *etc*. and is not relevant to the choice of reflections for refinement. *R*-factors based on *F*^2^ are statistically about twice as large as those based on *F*, and *R*- factors based on ALL data will be even larger.

### Fractional atomic coordinates and isotropic or equivalent isotropic displacement parameters (Å^2^)

**Table:**

	*x*	*y*	*z*	*U*_iso_*/*U*_eq_	
C1	0.3837 (3)	0.4533 (3)	0.1246 (6)	0.0332 (11)	
C2	0.3401 (3)	0.5067 (3)	0.0107 (6)	0.0365 (12)	
H2	0.2915	0.4874	−0.0540	0.044*	
C3	0.3245 (4)	0.6518 (4)	−0.1203 (8)	0.0556 (16)	
H3	0.2742	0.6365	−0.1840	0.067*	
C4	0.3558 (5)	0.7327 (4)	−0.1338 (9)	0.0653 (19)	
H4	0.3263	0.7728	−0.2047	0.078*	
C5	0.4321 (5)	0.7549 (4)	−0.0410 (8)	0.0629 (19)	
H5	0.4537	0.8096	−0.0539	0.076*	
C6	0.4753 (4)	0.6996 (4)	0.0668 (7)	0.0506 (15)	
H6	0.5258	0.7164	0.1281	0.061*	
C7	0.4575 (3)	0.4845 (3)	0.2190 (7)	0.0390 (13)	
H7	0.4861	0.4485	0.3000	0.047*	
C8	0.3683 (3)	0.5914 (3)	−0.0099 (7)	0.0388 (13)	
C9	0.4441 (3)	0.6161 (3)	0.0865 (7)	0.0378 (13)	
C10	0.3537 (3)	0.3649 (3)	0.1549 (6)	0.0281 (11)	
C11	0.0395 (3)	0.3680 (3)	0.3787 (6)	0.0351 (12)	
C12	0.0123 (3)	0.3445 (4)	0.5246 (7)	0.0441 (14)	
H12	0.0446	0.3078	0.5988	0.053*	
C13	−0.0952 (4)	0.3567 (5)	0.7191 (9)	0.077 (2)	
H13	−0.0651	0.3203	0.7973	0.093*	
C14	−0.1681 (5)	0.3917 (6)	0.7537 (11)	0.093 (3)	
H14	−0.1873	0.3802	0.8566	0.112*	
C15	−0.2145 (5)	0.4451 (5)	0.6353 (10)	0.076 (2)	
H15	−0.2649	0.4679	0.6596	0.091*	
C16	−0.1873 (4)	0.4638 (4)	0.4872 (9)	0.0564 (17)	
H16	−0.2188	0.4995	0.4098	0.068*	
C17	−0.0119 (4)	0.4216 (4)	0.2672 (7)	0.0466 (14)	
H17	0.0064	0.4359	0.1646	0.056*	
C18	−0.0649 (4)	0.3751 (4)	0.5657 (7)	0.0469 (14)	
C19	−0.1115 (4)	0.4298 (4)	0.4488 (7)	0.0439 (14)	
C20	0.1226 (3)	0.3383 (3)	0.3324 (7)	0.0358 (12)	
N1	0.4882 (3)	0.5620 (3)	0.1991 (6)	0.0419 (11)	
N2	−0.0843 (3)	0.4528 (3)	0.2993 (6)	0.0518 (13)	
O1	0.3147 (2)	0.3241 (2)	0.0321 (4)	0.0353 (8)	
O2	0.3661 (2)	0.3348 (2)	0.3017 (4)	0.0379 (9)	
O3	0.1346 (2)	0.3385 (3)	0.1807 (4)	0.0486 (10)	
O4	0.1760 (2)	0.3101 (2)	0.4499 (4)	0.0363 (8)	
O1W	0.3523 (2)	0.1106 (3)	0.1318 (5)	0.0446 (10)	
H2W	0.355 (3)	0.119 (4)	0.028 (2)	0.067*	
H1W	0.3984 (18)	0.093 (4)	0.182 (5)	0.067*	
O2W	0.1494 (3)	0.1094 (3)	0.3479 (5)	0.0535 (11)	
H3W	0.141 (4)	0.117 (3)	0.448 (3)	0.080*	
H4W	0.129 (4)	0.063 (2)	0.308 (6)	0.080*	
Sr1	0.25124 (3)	0.21557 (3)	0.23948 (6)	0.02880 (15)	

### Atomic displacement parameters (Å^2^)

**Table:**

	*U*^11^	*U*^22^	*U*^33^	*U*^12^	*U*^13^	*U*^23^
C1	0.037 (3)	0.030 (3)	0.033 (3)	−0.003 (2)	0.003 (2)	−0.006 (2)
C2	0.033 (3)	0.044 (3)	0.032 (3)	−0.009 (2)	0.004 (2)	−0.004 (2)
C3	0.062 (4)	0.054 (4)	0.048 (4)	−0.007 (3)	−0.006 (3)	0.010 (3)
C4	0.085 (5)	0.049 (4)	0.062 (4)	−0.004 (3)	0.011 (4)	0.022 (3)
C5	0.085 (5)	0.047 (4)	0.059 (4)	−0.022 (4)	0.020 (4)	0.004 (3)
C6	0.057 (4)	0.048 (4)	0.047 (4)	−0.013 (3)	0.009 (3)	−0.004 (3)
C7	0.037 (3)	0.042 (3)	0.036 (3)	−0.006 (2)	−0.004 (2)	−0.004 (2)
C8	0.040 (3)	0.036 (3)	0.042 (3)	−0.005 (2)	0.010 (3)	0.003 (2)
C9	0.041 (3)	0.034 (3)	0.039 (3)	−0.010 (2)	0.009 (3)	−0.005 (2)
C10	0.021 (3)	0.039 (3)	0.027 (3)	−0.002 (2)	0.014 (2)	−0.008 (2)
C11	0.031 (3)	0.042 (3)	0.031 (3)	0.005 (2)	−0.001 (2)	−0.004 (2)
C12	0.036 (3)	0.058 (4)	0.037 (3)	0.009 (3)	0.001 (2)	0.007 (3)
C13	0.056 (5)	0.120 (6)	0.060 (5)	0.024 (4)	0.023 (4)	0.025 (4)
C14	0.074 (6)	0.140 (8)	0.072 (6)	0.022 (5)	0.035 (5)	0.009 (5)
C15	0.049 (4)	0.095 (6)	0.086 (6)	0.016 (4)	0.024 (4)	−0.005 (5)
C16	0.044 (4)	0.061 (4)	0.067 (4)	0.016 (3)	0.016 (3)	−0.009 (3)
C17	0.048 (4)	0.055 (4)	0.037 (3)	0.015 (3)	0.006 (3)	0.009 (3)
C18	0.038 (3)	0.059 (4)	0.044 (4)	0.007 (3)	0.006 (3)	0.005 (3)
C19	0.038 (3)	0.044 (3)	0.051 (4)	0.001 (2)	0.008 (3)	−0.004 (3)
C20	0.037 (3)	0.035 (3)	0.034 (3)	0.004 (2)	0.000 (2)	−0.002 (2)
N1	0.039 (3)	0.046 (3)	0.040 (3)	−0.012 (2)	0.000 (2)	−0.005 (2)
N2	0.044 (3)	0.055 (3)	0.056 (3)	0.019 (2)	0.004 (2)	0.007 (2)
O1	0.042 (2)	0.0345 (19)	0.0281 (19)	−0.0058 (15)	−0.0005 (16)	−0.0059 (14)
O2	0.047 (2)	0.047 (2)	0.0189 (18)	−0.0094 (17)	0.0002 (15)	0.0023 (15)
O3	0.055 (3)	0.068 (3)	0.024 (2)	0.023 (2)	0.0086 (18)	0.0050 (17)
O4	0.033 (2)	0.051 (2)	0.0246 (18)	0.0092 (16)	0.0020 (15)	0.0017 (15)
O1W	0.044 (2)	0.056 (2)	0.033 (2)	0.0159 (19)	0.0027 (18)	0.0034 (18)
O2W	0.066 (3)	0.063 (3)	0.032 (2)	−0.029 (2)	0.009 (2)	−0.0082 (19)
Sr1	0.0294 (3)	0.0317 (2)	0.0244 (3)	−0.0002 (2)	−0.00020 (17)	0.00050 (19)

### Geometric parameters (Å, °)

**Table:**

C1—C2	1.357 (7)	C14—C15	1.398 (11)
C1—C7	1.409 (7)	C14—H14	0.9300
C1—C10	1.488 (7)	C15—C16	1.342 (9)
C2—C8	1.412 (7)	C15—H15	0.9300
C2—H2	0.9300	C16—C19	1.400 (8)
C3—C4	1.366 (8)	C16—H16	0.9300
C3—C8	1.412 (8)	C17—N2	1.320 (7)
C3—H3	0.9300	C17—H17	0.9300
C4—C5	1.394 (9)	C18—C19	1.406 (8)
C4—H4	0.9300	C19—N2	1.368 (7)
C5—C6	1.345 (9)	C20—O3	1.247 (6)
C5—H5	0.9300	C20—O4	1.265 (6)
C6—C9	1.411 (7)	O1—Sr1^i^	2.498 (3)
C6—H6	0.9300	O1—Sr1	2.658 (3)
C7—N1	1.322 (6)	O2—Sr1	2.624 (3)
C7—H7	0.9300	O3—Sr1	2.682 (4)
C8—C9	1.410 (7)	O4—Sr1^ii^	2.495 (3)
C9—N1	1.362 (7)	O4—Sr1	2.640 (3)
C10—O2	1.251 (6)	O1W—Sr1	2.534 (4)
C10—O1	1.263 (5)	O1W—H2W	0.840 (10)
C11—C12	1.344 (7)	O1W—H1W	0.841 (10)
C11—C17	1.407 (7)	O2W—Sr1	2.557 (4)
C11—C20	1.508 (7)	O2W—H3W	0.839 (10)
C12—C18	1.410 (8)	O2W—H4W	0.838 (10)
C12—H12	0.9300	Sr1—O4^i^	2.495 (3)
C13—C14	1.356 (10)	Sr1—O1^ii^	2.498 (3)
C13—C18	1.402 (8)	Sr1—H2W	2.93 (4)
C13—H13	0.9300		
			
C2—C1—C7	118.3 (5)	C20—O3—Sr1	91.3 (3)
C2—C1—C10	121.6 (5)	C20—O4—Sr1^ii^	160.4 (3)
C7—C1—C10	120.1 (5)	C20—O4—Sr1	92.8 (3)
C1—C2—C8	120.3 (5)	Sr1^ii^—O4—Sr1	106.78 (12)
C1—C2—H2	119.9	Sr1—O1W—H2W	110 (4)
C8—C2—H2	119.9	Sr1—O1W—H1W	128 (4)
C4—C3—C8	120.2 (6)	H2W—O1W—H1W	111.6 (14)
C4—C3—H3	119.9	Sr1—O2W—H3W	115 (4)
C8—C3—H3	119.9	Sr1—O2W—H4W	132 (3)
C3—C4—C5	119.8 (6)	H3W—O2W—H4W	111.9 (14)
C3—C4—H4	120.1	O4^i^—Sr1—O1^ii^	156.08 (11)
C5—C4—H4	120.1	O4^i^—Sr1—O1W	80.89 (12)
C6—C5—C4	121.9 (6)	O1^ii^—Sr1—O1W	87.25 (11)
C6—C5—H5	119.1	O4^i^—Sr1—O2W	87.22 (12)
C4—C5—H5	119.1	O1^ii^—Sr1—O2W	74.30 (13)
C5—C6—C9	119.9 (6)	O1W—Sr1—O2W	99.57 (14)
C5—C6—H6	120.1	O4^i^—Sr1—O2	122.32 (11)
C9—C6—H6	120.1	O1^ii^—Sr1—O2	78.73 (11)
N1—C7—C1	123.7 (5)	O1W—Sr1—O2	92.94 (13)
N1—C7—H7	118.2	O2W—Sr1—O2	149.55 (11)
C1—C7—H7	118.2	O4^i^—Sr1—O4	117.83 (13)
C9—C8—C2	117.4 (5)	O1^ii^—Sr1—O4	73.29 (10)
C9—C8—C3	119.1 (5)	O1W—Sr1—O4	160.47 (11)
C2—C8—C3	123.5 (5)	O2W—Sr1—O4	77.18 (13)
N1—C9—C8	122.1 (5)	O2—Sr1—O4	81.79 (11)
N1—C9—C6	118.7 (5)	O4^i^—Sr1—O1	73.02 (10)
C8—C9—C6	119.2 (5)	O1^ii^—Sr1—O1	126.30 (13)
O2—C10—O1	122.6 (4)	O1W—Sr1—O1	83.32 (12)
O2—C10—C1	118.7 (4)	O2W—Sr1—O1	159.40 (12)
O1—C10—C1	118.6 (4)	O2—Sr1—O1	49.34 (10)
C12—C11—C17	118.4 (5)	O4—Sr1—O1	106.60 (10)
C12—C11—C20	121.8 (5)	O4^i^—Sr1—O3	72.94 (12)
C17—C11—C20	119.8 (5)	O1^ii^—Sr1—O3	122.19 (11)
C11—C12—C18	120.3 (5)	O1W—Sr1—O3	150.27 (11)
C11—C12—H12	119.9	O2W—Sr1—O3	93.09 (14)
C18—C12—H12	119.9	O2—Sr1—O3	89.40 (12)
C14—C13—C18	120.2 (7)	O4—Sr1—O3	48.96 (10)
C14—C13—H13	119.9	O1—Sr1—O3	75.85 (11)
C18—C13—H13	119.9	O4^i^—Sr1—Sr1^ii^	150.83 (8)
C13—C14—C15	120.3 (7)	O1^ii^—Sr1—Sr1^ii^	38.27 (8)
C13—C14—H14	119.8	O1W—Sr1—Sr1^ii^	125.06 (9)
C15—C14—H14	119.8	O2W—Sr1—Sr1^ii^	76.24 (9)
C16—C15—C14	120.9 (7)	O2—Sr1—Sr1^ii^	73.85 (8)
C16—C15—H15	119.5	O4—Sr1—Sr1^ii^	35.41 (7)
C14—C15—H15	119.5	O1—Sr1—Sr1^ii^	118.86 (7)
C15—C16—C19	120.2 (6)	O3—Sr1—Sr1^ii^	83.99 (8)
C15—C16—H16	119.9	O4^i^—Sr1—Sr1^i^	37.81 (8)
C19—C16—H16	119.9	O1^ii^—Sr1—Sr1^i^	156.01 (8)
N2—C17—C11	124.0 (5)	O1W—Sr1—Sr1^i^	76.17 (9)
N2—C17—H17	118.0	O2W—Sr1—Sr1^i^	125.03 (9)
C11—C17—H17	118.0	O2—Sr1—Sr1^i^	84.84 (7)
C13—C18—C19	118.9 (6)	O4—Sr1—Sr1^i^	121.68 (7)
C13—C18—C12	123.4 (6)	O1—Sr1—Sr1^i^	35.59 (7)
C19—C18—C12	117.6 (5)	O3—Sr1—Sr1^i^	74.54 (8)
N2—C19—C16	118.5 (6)	Sr1^ii^—Sr1—Sr1^i^	149.85 (2)
N2—C19—C18	122.0 (5)	O4^i^—Sr1—H2W	68.4 (7)
C16—C19—C18	119.4 (6)	O1^ii^—Sr1—H2W	102.4 (5)
O3—C20—O4	122.8 (5)	O1W—Sr1—H2W	15.7 (6)
O3—C20—C11	119.3 (5)	O2W—Sr1—H2W	107.5 (11)
O4—C20—C11	117.8 (5)	O2—Sr1—H2W	91.7 (11)
C7—N1—C9	118.1 (4)	O4—Sr1—H2W	172.8 (10)
C17—N2—C19	117.6 (5)	O1—Sr1—H2W	71.0 (10)
C10—O1—Sr1^i^	161.6 (3)	O3—Sr1—H2W	134.7 (6)
C10—O1—Sr1	91.8 (3)	Sr1^ii^—Sr1—H2W	139.3 (6)
Sr1^i^—O1—Sr1	106.14 (12)	Sr1^i^—Sr1—H2W	60.5 (6)
C10—O2—Sr1	93.7 (3)		

Symmetry codes: (i) *x*, −*y*+1/2, *z*−1/2; (ii) *x*, −*y*+1/2, *z*+1/2.

### Hydrogen-bond geometry (Å, °)

**Table:**

*D*—H···*A*	*D*—H	H···*A*	*D*···*A*	*D*—H···*A*
O1W—H2W···O2^i^	0.84 (1)	1.97 (2)	2.798 (5)	168 (6)
O1W—H1W···N1^iii^	0.84 (1)	2.01 (1)	2.846 (6)	175 (6)
O2W—H3W···O3^ii^	0.84 (1)	1.99 (2)	2.810 (5)	166 (5)
O2W—H4W···N2^iv^	0.84 (1)	2.01 (1)	2.846 (6)	176 (5)

Symmetry codes: (i) *x*, −*y*+1/2, *z*−1/2; (iii) −*x*+1, *y*−1/2, −*z*+1/2; (ii) *x*, −*y*+1/2, *z*+1/2; (iv) −*x*, *y*−1/2, −*z*+1/2.

## References

[bb1] Bruker (2001). *SADABS* Bruker AXS Inc., Madison, Wisconsin, USA.

[bb2] Bruker (2002). *SAINT* Bruker AXS Inc., Madison, Wisconsin, USA.

[bb3] Bruker (2004). *APEX2* Bruker AXS Inc., Madison, Wisconsin, USA.

[bb4] Haendler, H. M. (1986). *Acta Cryst.* C**42**, 147–149.

[bb5] Haendler, H. M. (1996). *Acta Cryst.* C**52**, 801–803.

[bb6] Hu, S., Zhang, S.-H. & Zeng, M.-H. (2007). *Acta Cryst.* E**63**, m2565.

[bb7] Martell, A. E. & Smith, R. M. (1974). *Critical Stability Constants*, Vol. 1, pp. 78, 372; Vol. 2, p. 219. New York: Plenum Press.

[bb8] Miao, D.-L., Li, S.-J., Song, W.-D., Liu, J.-H. & Li, X.-F. (2010). *Acta Cryst.* E**66**, m1441–m1442. 10.1107/S1600536810039401 PMC3009206 21588864

[bb9] Odoko, M., Muranishi, Y. & Okabe, N. (2001). *Acta Cryst.* E**57**, m267–m269.

[bb10] Okabe, N. & Koizumi, M. (1997). *Acta Cryst.* C**53**, 852–854.

[bb11] Okabe, N. & Makino, T. (1998). *Acta Cryst.* C**54**, 1279–1280.

[bb12] Okabe, N. & Makino, T. (1999). *Acta Cryst.* C**55**, 300–302.

[bb13] Okabe, N. & Muranishi, Y. (2002). *Acta Cryst.* E**58**, m287–m289.

[bb14] Okabe, N. & Muranishi, Y. (2003*a*). *Acta Cryst.* E**59**, m220–m222. 10.1107/s0108270103008722 12794330

[bb15] Okabe, N. & Muranishi, Y. (2003*b*). *Acta Cryst.* E**59**, m244–m246. 10.1107/s0108270103008722 12794330

[bb16] Sheldrick, G. M. (2008). *Acta Cryst.* A**64**, 112–122. 10.1107/S0108767307043930 18156677

[bb17] Zevaco, T. A., Gorls, H. & Dinjus, E. (1998). *Inorg. Chim. Acta*, **269**, 283–286.

